# Supplementary material for: Enhancement of Vaccine-Induced T-Cell Responses by PD-L1 Blockade in Calves
Source: Vaccines (Basel). 2023 Mar 1;11(3):559. doi: 10.3390/vaccines11030559 (PMC10051159; doi:10.3390/vaccines11030559)
Supplement: Supplementary file 1 [file vaccines-11-00559-s001.zip › vaccines-2232578-supplementary.pptx]

## Slide 1
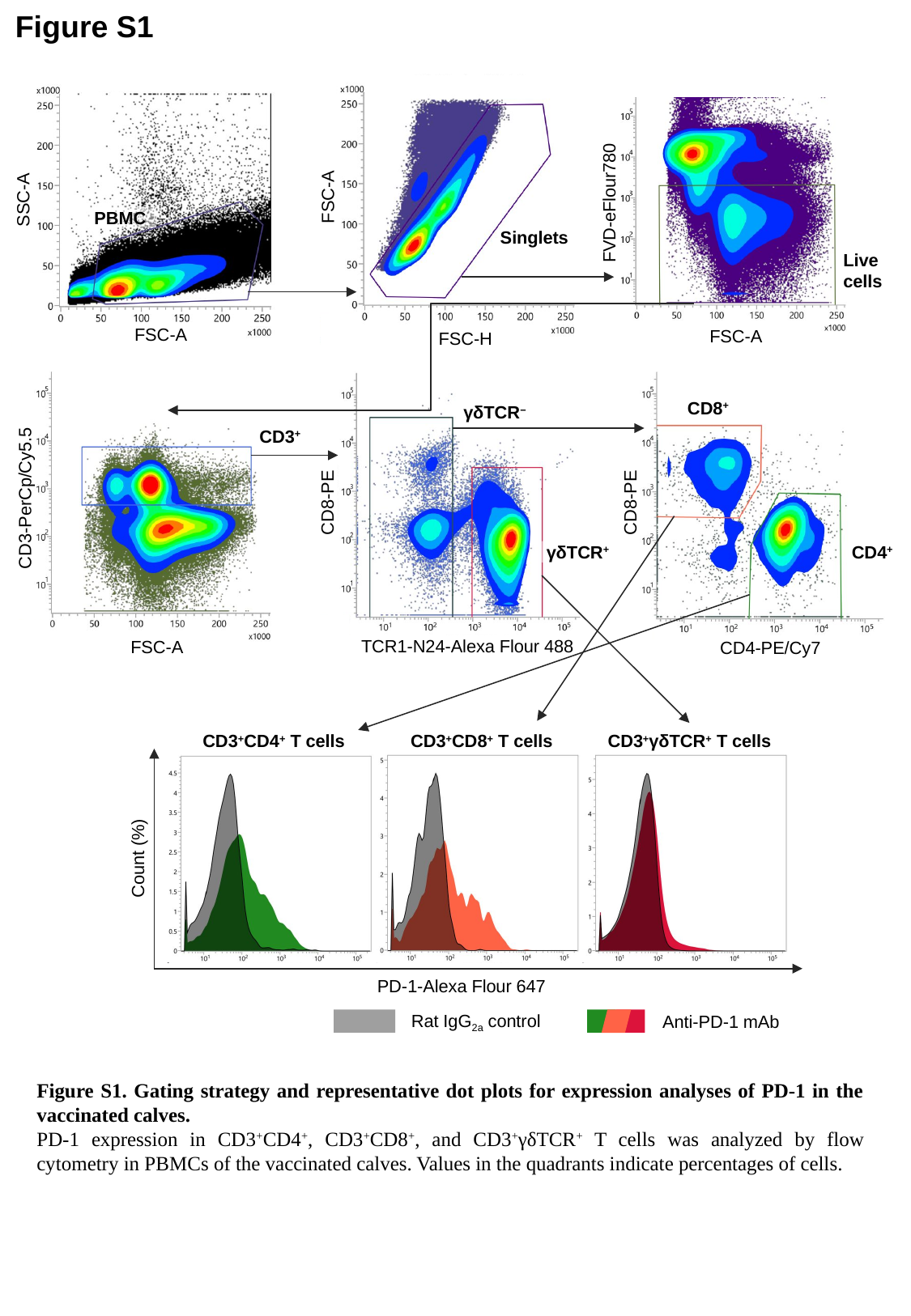

Figure S1
PBMC
Live
cells
CD8+
γδTCR−
CD3+
γδTCR+
CD4+
FSC-A
SSC-A
FVD-eFlour780
Singlets
FSC-A
FSC-A
FSC-H
CD3-PerCp/Cy5.5
CD8-PE
CD8-PE
TCR1-N24-Alexa Flour 488
FSC-A
CD4-PE/Cy7
CD3+CD4+ T cells
CD3+CD8+ T cells
CD3+γδTCR+ T cells
Count (%)
PD-1-Alexa Flour 647
Rat IgG2a control
Anti-PD-1 mAb
Figure S1. Gating strategy and representative dot plots for expression analyses of PD-1 in the vaccinated calves.
PD-1 expression in CD3+CD4+, CD3+CD8+, and CD3+γδTCR+ T cells was analyzed by flow cytometry in PBMCs of the vaccinated calves. Values in the quadrants indicate percentages of cells.

## Slide 2
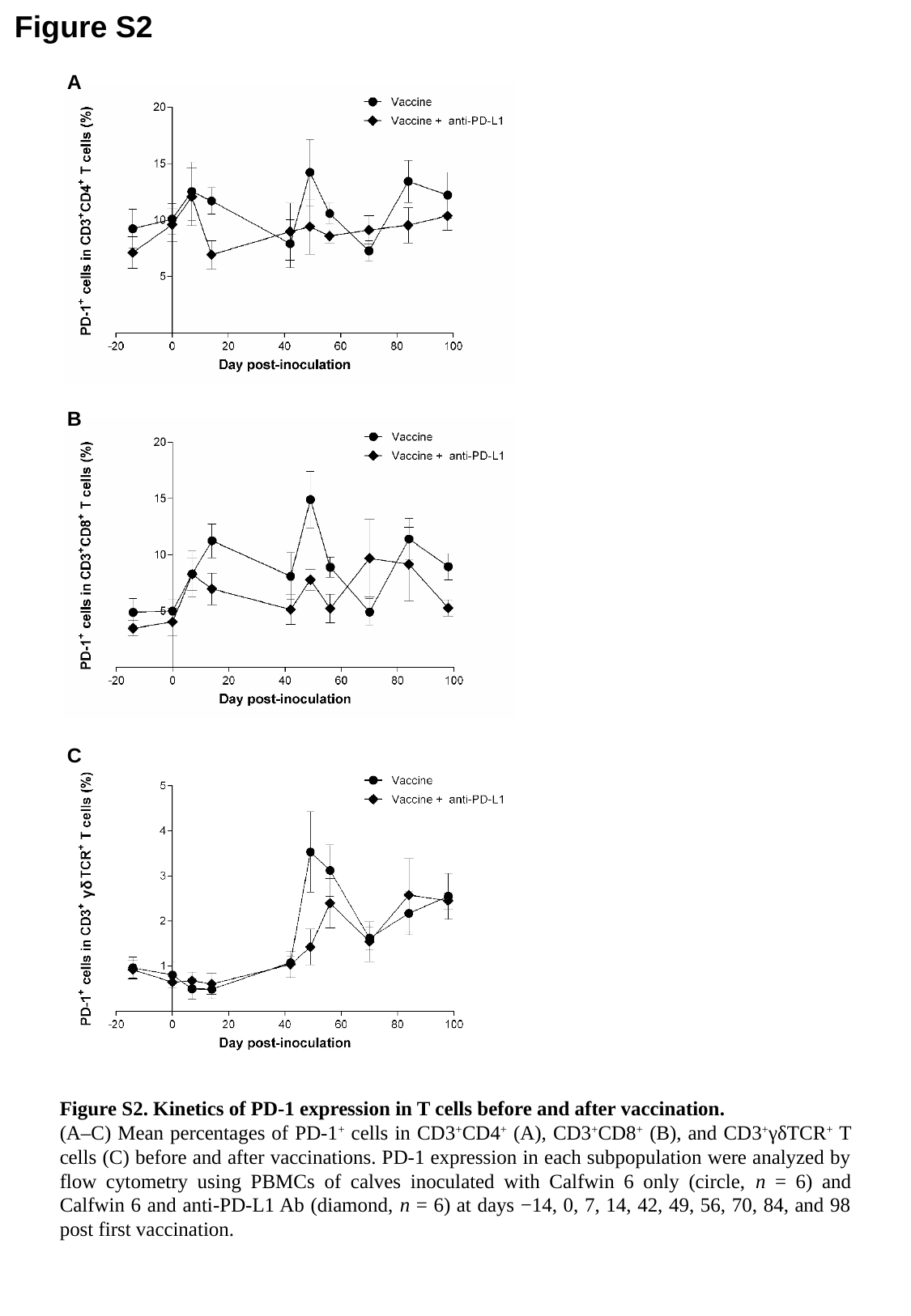

Figure S2
A
B
C
γδ
Figure S2. Kinetics of PD-1 expression in T cells before and after vaccination.
(A–C) Mean percentages of PD-1+ cells in CD3+CD4+ (A), CD3+CD8+ (B), and CD3+γδTCR+ T cells (C) before and after vaccinations. PD-1 expression in each subpopulation were analyzed by flow cytometry using PBMCs of calves inoculated with Calfwin 6 only (circle, n = 6) and Calfwin 6 and anti-PD-L1 Ab (diamond, n = 6) at days −14, 0, 7, 14, 42, 49, 56, 70, 84, and 98 post first vaccination.

## Slide 3
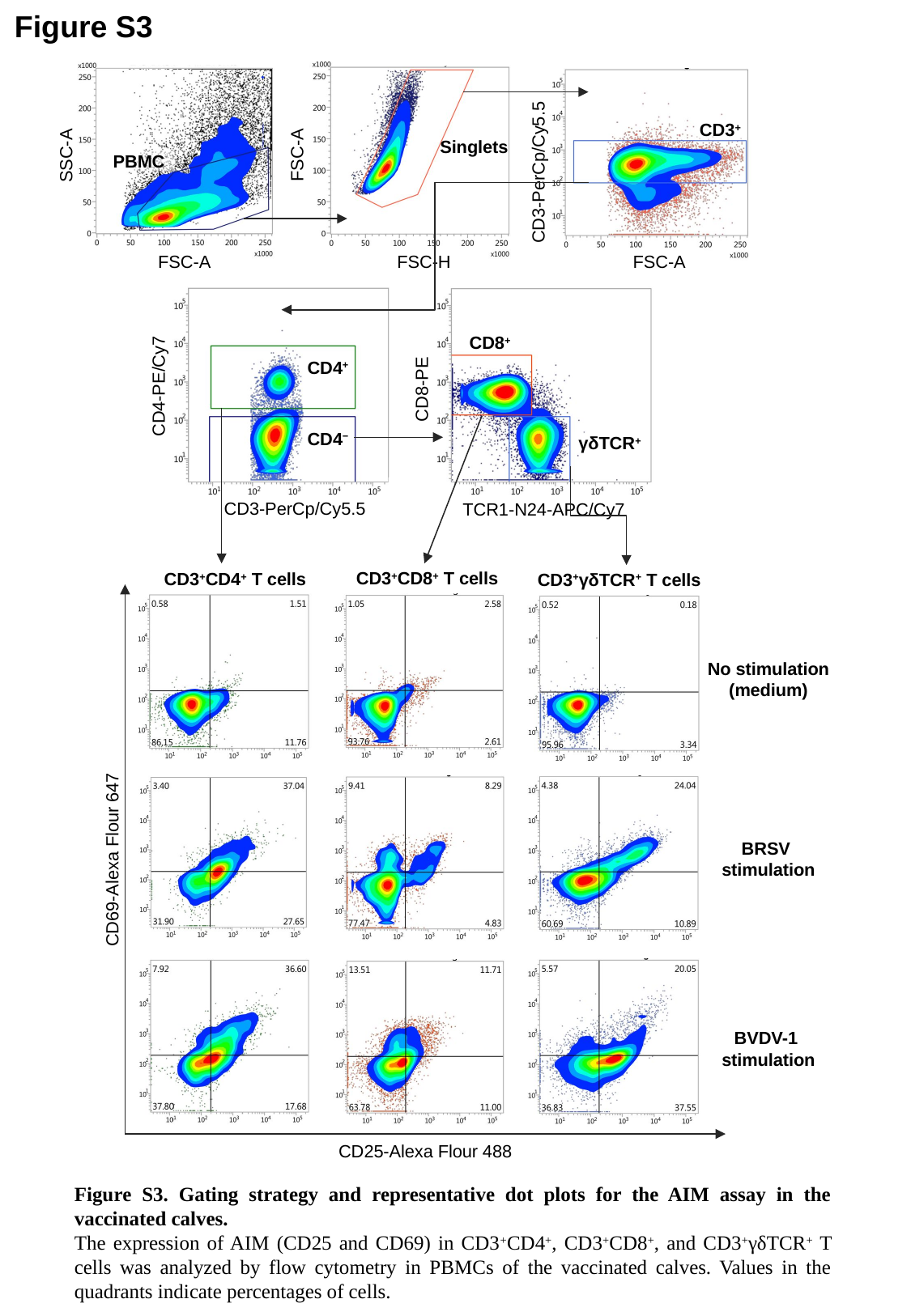

Figure S3
SSC-A
PBMC
FSC-A
Singlets
FSC-A
FSC-H
CD3+
CD3-PerCp/Cy5.5
FSC-A
CD4+
CD4-PE/Cy7
CD4−
CD3-PerCp/Cy5.5
CD8+
CD8-PE
γδTCR+
TCR1-N24-APC/Cy7
CD3+CD8+ T cells
CD3+CD4+ T cells
CD3+γδTCR+ T cells
CD25-Alexa Flour 488
CD69-Alexa Flour 647
No stimulation
(medium)
BRSV
stimulation
BVDV-1
stimulation
Figure S3. Gating strategy and representative dot plots for the AIM assay in the vaccinated calves.
The expression of AIM (CD25 and CD69) in CD3+CD4+, CD3+CD8+, and CD3+γδTCR+ T cells was analyzed by flow cytometry in PBMCs of the vaccinated calves. Values in the quadrants indicate percentages of cells.

## Slide 4
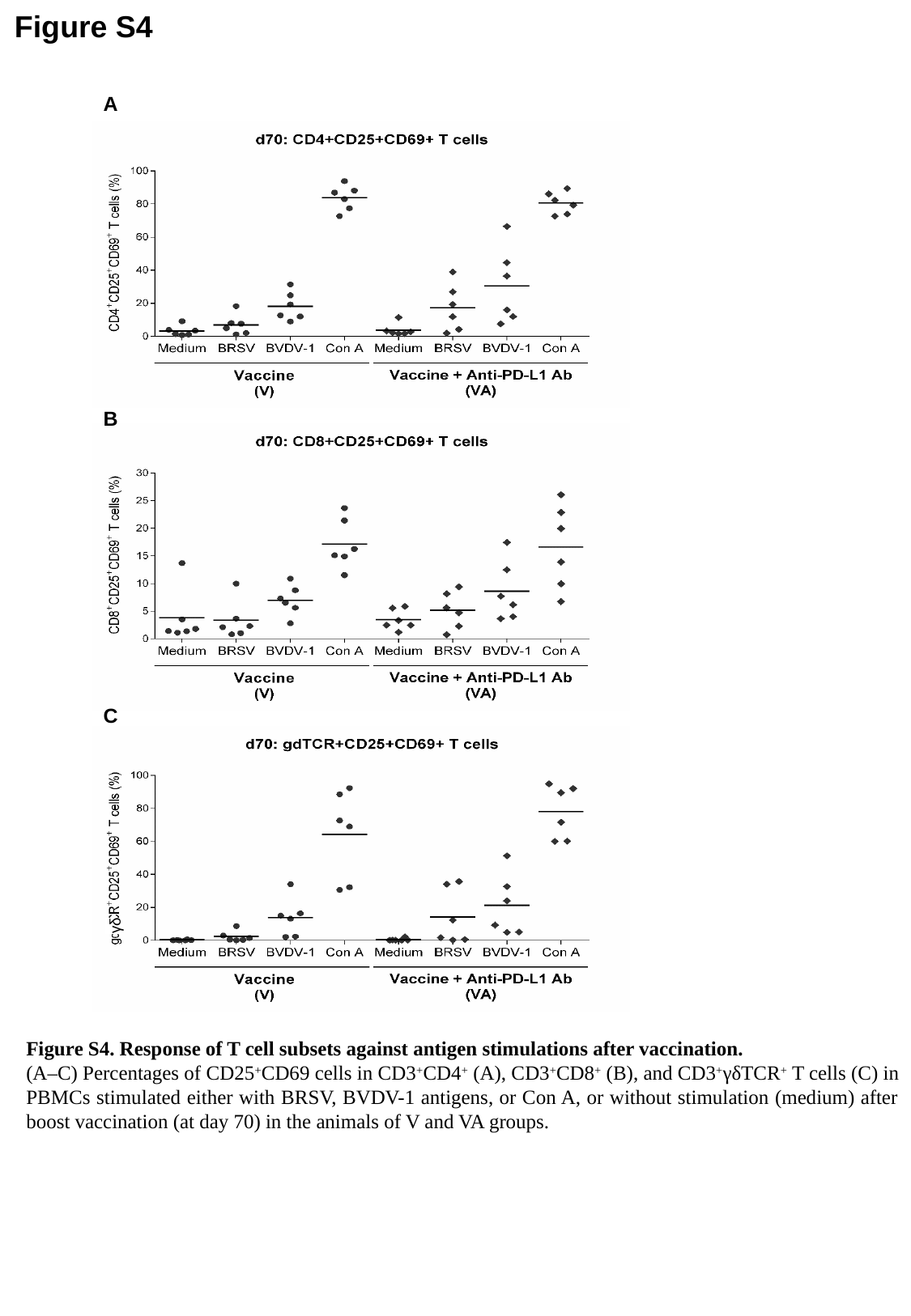

Figure S4
A
B
C
γδ
Figure S4. Response of T cell subsets against antigen stimulations after vaccination.
(A–C) Percentages of CD25+CD69 cells in CD3+CD4+ (A), CD3+CD8+ (B), and CD3+γδTCR+ T cells (C) in PBMCs stimulated either with BRSV, BVDV-1 antigens, or Con A, or without stimulation (medium) after boost vaccination (at day 70) in the animals of V and VA groups.

## Slide 5
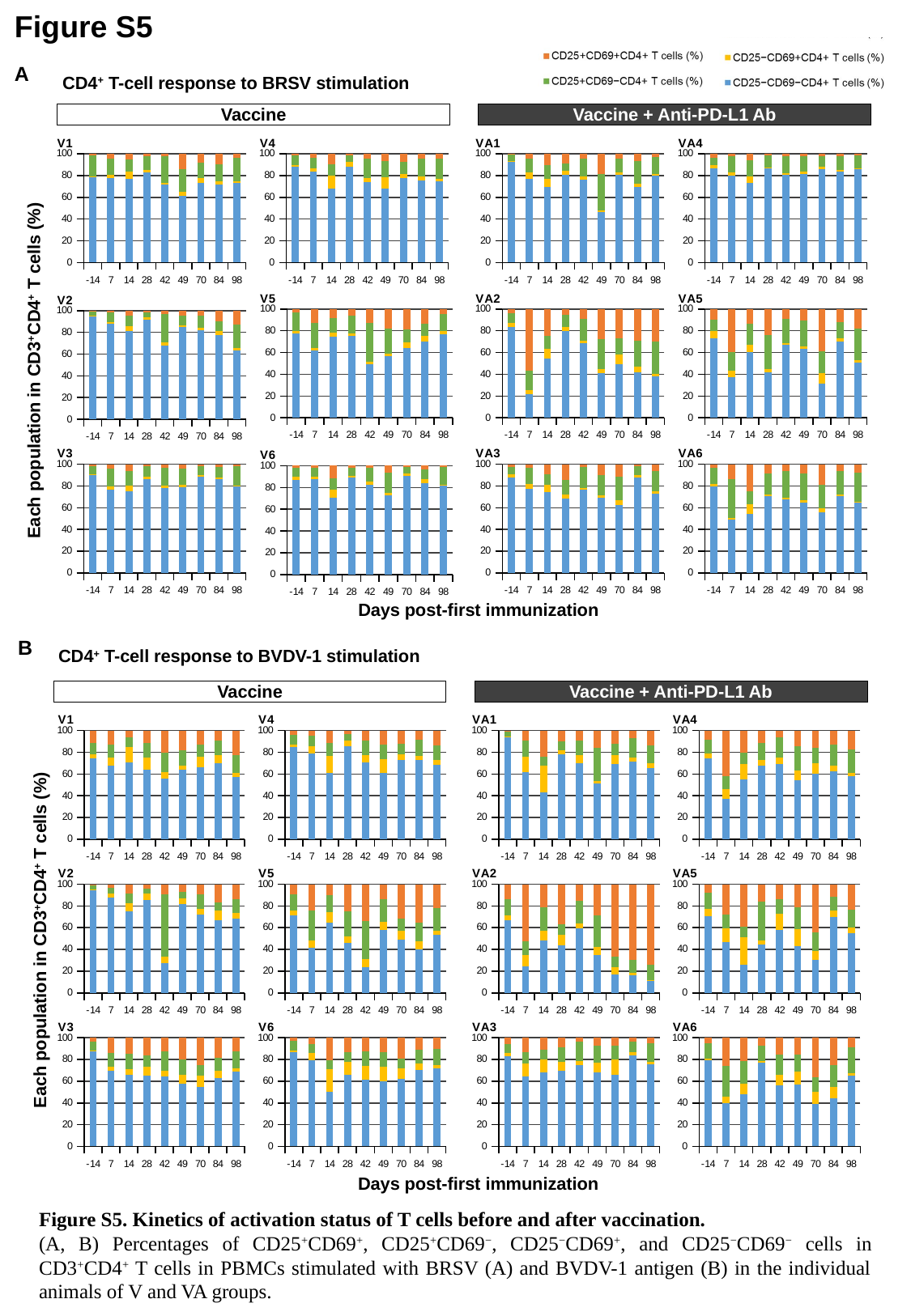

Figure S5
A
CD4+ T-cell response to BRSV stimulation
Vaccine + Anti-PD-L1 Ab
Vaccine
### Chart:
| Category | CD25−CD69−CD4+ T cells (%) | CD25−CD69+CD4+ T cells (%) | CD25+CD69−CD4+ T cells (%) | CD25+CD69+CD4+ T cells (%) |
|---|---|---|---|---|
| -14 | 78.39333333333333 | 0.73 | 19.3 | 1.5766666666666669 |
| 7 | 77.37666666666667 | 3.473333333333333 | 14.726666666666667 | 4.423333333333333 |
| 14 | 76.92999999999999 | 6.676666666666667 | 11.453333333333333 | 4.94 |
| 28 | 83.29 | 1.7933333333333337 | 12.973333333333334 | 1.9433333333333334 |
| 42 | 71.83 | 1.8066666666666666 | 24.096666666666664 | 2.2666666666666666 |
| 49 | 61.63 | 3.2600000000000002 | 21.320000000000004 | 13.79 |
| 70 | 73.54666666666667 | 3.8966666666666665 | 14.450000000000001 | 8.106666666666667 |
| 84 | 71.43666666666667 | 3.25 | 15.936666666666666 | 9.376666666666667 |
| 98 | 73.25666666666667 | 1.2633333333333334 | 22.166666666666668 | 3.313333333333333 |
### Chart:
| Category | CD25−CD69−CD4+ T cells (%) | CD25−CD69+CD4+ T cells (%) | CD25+CD69−CD4+ T cells (%) | CD25+CD69+CD4+ T cells (%) |
|---|---|---|---|---|
| -14 | 87.98333333333333 | 1.6566666666666665 | 8.86 | 1.5 |
| 7 | 84.02666666666669 | 2.3233333333333333 | 10.27 | 3.3800000000000003 |
| 14 | 68.10333333333334 | 11.976666666666667 | 10.333333333333334 | 9.586666666666666 |
| 28 | 88.17999999999999 | 4.746666666666667 | 5.386666666666667 | 1.6866666666666665 |
| 42 | 74.21666666666668 | 3.1999999999999997 | 18.333333333333332 | 4.25 |
| 49 | 67.71333333333334 | 10.943333333333333 | 14.67 | 6.673333333333335 |
| 70 | 78.01333333333334 | 3.2933333333333334 | 11.016666666666666 | 7.676666666666667 |
| 84 | 75.56666666666668 | 3.6633333333333336 | 16.33666666666667 | 4.433333333333334 |
| 98 | 74.92666666666666 | 2.0100000000000002 | 18.766666666666666 | 4.296666666666667 |
### Chart:
| Category | CD25−CD69−CD4+ T cells (%) | CD25−CD69+CD4+ T cells (%) | CD25+CD69−CD4+ T cells (%) | CD25+CD69+CD4+ T cells (%) |
|---|---|---|---|---|
| -14 | 92.64666666666666 | 1.0166666666666666 | 5.46 | 0.8766666666666666 |
| 7 | 77.26333333333334 | 5.390000000000001 | 12.99 | 4.3566666666666665 |
| 14 | 69.32666666666665 | 7.56 | 12.466666666666667 | 10.646666666666667 |
| 28 | 80.53333333333333 | 3.5399999999999996 | 7.37 | 8.556666666666667 |
| 42 | 76.43666666666667 | 2.5733333333333337 | 16.3 | 4.69 |
| 49 | 46.093333333333334 | 1.8166666666666667 | 33.20333333333333 | 18.886666666666667 |
| 70 | 80.58666666666666 | 2.2133333333333334 | 12.93 | 4.27 |
| 84 | 69.74 | 3.016666666666667 | 20.779999999999998 | 6.463333333333334 |
| 98 | 80.05666666666666 | 1.29 | 15.526666666666666 | 3.1266666666666665 |
### Chart:
| Category | CD25−CD69−CD4+ T cells (%) | CD25−CD69+CD4+ T cells (%) | CD25+CD69−CD4+ T cells (%) | CD25+CD69+CD4+ T cells (%) |
|---|---|---|---|---|
| -14 | 86.64333333333333 | 3.09 | 6.513333333333333 | 3.753333333333334 |
| 7 | 80.0 | 3.14 | 14.746666666666664 | 2.1133333333333333 |
| 14 | 73.58333333333333 | 5.543333333333333 | 15.246666666666664 | 5.626666666666666 |
| 28 | 86.42333333333333 | 1.2533333333333332 | 10.916666666666666 | 1.406666666666667 |
| 42 | 81.05666666666666 | 1.1466666666666667 | 15.589999999999998 | 2.2066666666666666 |
| 49 | 81.10333333333332 | 2.4066666666666667 | 14.203333333333333 | 2.2866666666666666 |
| 70 | 85.75333333333333 | 2.15 | 10.173333333333334 | 1.9233333333333331 |
| 84 | 83.60666666666667 | 1.4466666666666665 | 12.843333333333334 | 2.1033333333333335 |
| 98 | 85.62 | 1.03 | 12.056666666666667 | 1.2933333333333332 |
### Chart:
| Category | CD25−CD69−CD4+ T cells (%) | CD25−CD69+CD4+ T cells (%) | CD25+CD69−CD4+ T cells (%) | CD25+CD69+CD4+ T cells (%) |
|---|---|---|---|---|
| -14 | 77.75333333333333 | 2.2533333333333334 | 16.886666666666667 | 3.106666666666667 |
| 7 | 61.873333333333335 | 2.3766666666666665 | 23.13 | 12.62 |
| 14 | 74.75 | 3.7600000000000002 | 13.536666666666667 | 7.953333333333333 |
| 28 | 75.44 | 1.9833333333333332 | 16.213333333333335 | 6.363333333333333 |
| 42 | 49.626666666666665 | 2.0533333333333332 | 35.52 | 12.800000000000002 |
| 49 | 56.873333333333335 | 2.3433333333333333 | 23.016666666666666 | 17.76666666666667 |
| 70 | 64.03333333333335 | 5.206666666666667 | 12.393333333333333 | 18.366666666666667 |
| 84 | 70.16666666666667 | 4.916666666666667 | 11.110000000000001 | 13.806666666666667 |
| 98 | 77.02666666666667 | 2.5033333333333334 | 15.589999999999998 | 4.88 |
### Chart:
| Category | CD25−CD69−CD4+ T cells (%) | CD25−CD69+CD4+ T cells (%) | CD25+CD69−CD4+ T cells (%) | CD25+CD69+CD4+ T cells (%) |
|---|---|---|---|---|
| -14 | 83.53333333333335 | 3.91 | 8.49 | 4.066666666666667 |
| 7 | 21.396666666666665 | 4.283333333333334 | 17.930000000000003 | 56.39000000000001 |
| 14 | 54.45333333333334 | 9.093333333333332 | 11.719999999999999 | 24.733333333333334 |
| 28 | 79.53333333333333 | 4.216666666666666 | 10.753333333333336 | 5.496666666666666 |
| 42 | 68.25666666666666 | 2.4 | 20.393333333333334 | 8.950000000000001 |
| 49 | 41.03333333333334 | 3.4766666666666666 | 27.95 | 27.540000000000003 |
| 70 | 49.64333333333334 | 8.276666666666666 | 15.063333333333333 | 27.016666666666666 |
| 84 | 41.65 | 5.596666666666667 | 23.3 | 29.453333333333333 |
| 98 | 38.02 | 2.41 | 30.036666666666665 | 29.533333333333335 |
### Chart:
| Category | CD25−CD69−CD4+ T cells (%) | CD25−CD69+CD4+ T cells (%) | CD25+CD69−CD4+ T cells (%) | CD25+CD69+CD4+ T cells (%) |
|---|---|---|---|---|
| -14 | 72.82333333333332 | 6.826666666666667 | 10.38 | 9.97 |
| 7 | 37.36666666666667 | 5.73 | 17.209999999999997 | 39.693333333333335 |
| 14 | 60.643333333333324 | 6.496666666666667 | 19.323333333333334 | 13.536666666666667 |
| 28 | 41.49666666666667 | 3.103333333333333 | 31.159999999999997 | 24.24 |
| 42 | 66.77333333333333 | 1.8633333333333333 | 22.573333333333334 | 8.79 |
| 49 | 63.15666666666666 | 2.31 | 23.703333333333333 | 10.83 |
| 70 | 31.396666666666665 | 9.396666666666667 | 20.296666666666667 | 38.910000000000004 |
| 84 | 70.4 | 2.846666666666667 | 14.933333333333332 | 11.82 |
| 98 | 50.836666666666666 | 2.0533333333333332 | 28.776666666666667 | 18.333333333333332 |
### Chart:
| Category | CD25−CD69−CD4+ T cells (%) | CD25−CD69+CD4+ T cells (%) | CD25+CD69−CD4+ T cells (%) | CD25+CD69+CD4+ T cells (%) |
|---|---|---|---|---|
| -14 | 94.81333333333333 | 0.5 | 4.116666666666666 | 0.57 |
| 7 | 88.08999999999999 | 1.29 | 8.6 | 2.02 |
| 14 | 81.13666666666667 | 4.336666666666667 | 9.926666666666666 | 4.6000000000000005 |
| 28 | 91.64666666666666 | 1.8833333333333335 | 4.993333333333333 | 1.4766666666666666 |
| 42 | 68.00999999999999 | 2.623333333333333 | 25.856666666666666 | 3.5100000000000002 |
| 49 | 84.76666666666665 | 1.8866666666666667 | 8.680000000000001 | 4.666666666666667 |
| 70 | 81.78 | 2.5 | 10.733333333333334 | 4.986666666666666 |
| 84 | 77.56333333333333 | 3.7099999999999995 | 8.92 | 9.806666666666667 |
| 98 | 63.28666666666667 | 1.9566666666666663 | 22.26 | 12.496666666666664 |Each population in CD3+CD4+ T cells (%)
### Chart:
| Category | CD25−CD69−CD4+ T cells (%) | CD25−CD69+CD4+ T cells (%) | CD25+CD69−CD4+ T cells (%) | CD25+CD69+CD4+ T cells (%) |
|---|---|---|---|---|
| -14 | 90.12333333333333 | 0.6666666666666666 | 7.62 | 1.5899999999999999 |
| 7 | 76.82000000000001 | 2.69 | 16.176666666666666 | 4.3133333333333335 |
| 14 | 75.39 | 5.1499999999999995 | 12.943333333333333 | 6.516666666666667 |
| 28 | 86.49000000000001 | 2.04 | 9.616666666666667 | 1.8533333333333335 |
| 42 | 78.14999999999999 | 2.2066666666666666 | 16.543333333333333 | 3.1 |
| 49 | 78.54333333333332 | 2.48 | 14.623333333333335 | 4.353333333333333 |
| 70 | 88.14666666666666 | 1.6366666666666667 | 8.133333333333335 | 2.0833333333333335 |
| 84 | 86.01666666666667 | 1.7866666666666668 | 9.463333333333333 | 2.733333333333333 |
| 98 | 79.78666666666666 | 0.63 | 17.933333333333334 | 1.6500000000000004 |
### Chart:
| Category | CD25−CD69−CD4+ T cells (%) | CD25−CD69+CD4+ T cells (%) | CD25+CD69−CD4+ T cells (%) | CD25+CD69+CD4+ T cells (%) |
|---|---|---|---|---|
| -14 | 87.45666666666666 | 3.27 | 6.656666666666666 | 2.6166666666666667 |
| 7 | 77.46999999999998 | 4.506666666666667 | 14.43 | 3.5933333333333337 |
| 14 | 74.29666666666668 | 6.386666666666667 | 10.23 | 9.086666666666668 |
| 28 | 68.74333333333333 | 3.6666666666666665 | 13.283333333333333 | 14.306666666666667 |
| 42 | 76.80333333333334 | 1.5833333333333333 | 18.796666666666667 | 2.8166666666666664 |
| 49 | 68.96666666666665 | 2.61 | 18.106666666666666 | 10.316666666666666 |
| 70 | 62.21 | 4.796666666666667 | 21.133333333333336 | 11.86 |
| 84 | 87.85666666666668 | 2.146666666666667 | 7.919999999999999 | 2.0766666666666667 |
| 98 | 72.97333333333333 | 1.8066666666666666 | 19.173333333333336 | 6.046666666666667 |
### Chart:
| Category | CD25−CD69−CD4+ T cells (%) | CD25−CD69+CD4+ T cells (%) | CD25+CD69−CD4+ T cells (%) | CD25+CD69+CD4+ T cells (%) |
|---|---|---|---|---|
| -14 | 79.60666666666667 | 2.266666666666667 | 14.726666666666667 | 3.4 |
| 7 | 49.06 | 1.8066666666666666 | 35.42000000000001 | 13.713333333333333 |
| 14 | 54.45333333333334 | 9.093333333333332 | 11.719999999999999 | 24.733333333333334 |
| 28 | 70.44 | 1.8333333333333333 | 19.323333333333334 | 8.403333333333334 |
| 42 | 67.46666666666665 | 1.8699999999999999 | 24.063333333333333 | 6.6000000000000005 |
| 49 | 64.88333333333334 | 2.04 | 24.73333333333333 | 8.343333333333332 |
| 70 | 55.449999999999996 | 3.9766666666666666 | 21.256666666666668 | 19.316666666666666 |
| 84 | 70.96333333333332 | 1.37 | 21.173333333333336 | 6.493333333333333 |
| 98 | 64.60333333333334 | 0.85 | 27.08 | 7.466666666666666 |
### Chart:
| Category | CD25−CD69−CD4+ T cells (%) | CD25−CD69+CD4+ T cells (%) | CD25+CD69−CD4+ T cells (%) | CD25+CD69+CD4+ T cells (%) |
|---|---|---|---|---|
| -14 | 86.86666666666667 | 2.6799999999999997 | 8.423333333333334 | 2.03 |
| 7 | 87.45666666666666 | 2.6 | 8.146666666666667 | 1.7966666666666669 |
| 14 | 70.57666666666667 | 7.303333333333332 | 10.203333333333333 | 11.916666666666666 |
| 28 | 89.14 | 1.8533333333333335 | 7.05 | 1.9566666666666663 |
| 42 | 82.74666666666666 | 2.84 | 12.436666666666667 | 1.9766666666666666 |
| 49 | 72.88000000000001 | 2.5566666666666666 | 18.55666666666667 | 6.006666666666668 |
| 70 | 90.77666666666666 | 1.89 | 6.096666666666667 | 1.2366666666666666 |
| 84 | 83.69000000000001 | 3.7933333333333334 | 9.28 | 3.236666666666667 |
| 98 | 81.42 | 1.04 | 16.34 | 1.2 |Days post-first immunization
B
CD4+ T-cell response to BVDV-1 stimulation
Vaccine + Anti-PD-L1 Ab
Vaccine
### Chart:
| Category | CD25−CD69−CD4+ T cells (%) | CD25−CD69+CD4+ T cells (%) | CD25+CD69−CD4+ T cells (%) | CD25+CD69+CD4+ T cells (%) |
|---|---|---|---|---|
| -14 | 74.30000000000001 | 3.9200000000000004 | 10.026666666666667 | 11.753333333333336 |
| 7 | 67.99000000000001 | 6.866666666666667 | 12.306666666666667 | 12.836666666666666 |
| 14 | 70.95 | 13.796666666666667 | 8.963333333333333 | 6.29 |
| 28 | 63.75666666666666 | 11.173333333333332 | 13.756666666666668 | 11.313333333333333 |
| 42 | 55.54333333333333 | 5.91 | 18.08 | 20.466666666666665 |
| 49 | 63.94666666666666 | 3.7100000000000004 | 14.229999999999999 | 18.113333333333333 |
| 70 | 66.12333333333333 | 10.0 | 11.156666666666666 | 12.719999999999999 |
| 84 | 70.01333333333334 | 7.346666666666667 | 13.433333333333332 | 9.206666666666665 |
| 98 | 57.64000000000001 | 3.313333333333333 | 16.256666666666664 | 22.790000000000003 |
### Chart:
| Category | CD25−CD69−CD4+ T cells (%) | CD25−CD69+CD4+ T cells (%) | CD25+CD69−CD4+ T cells (%) | CD25+CD69+CD4+ T cells (%) |
|---|---|---|---|---|
| -14 | 85.14 | 1.9766666666666666 | 9.24 | 3.643333333333333 |
| 7 | 78.93333333333334 | 6.38 | 10.11 | 4.576666666666667 |
| 14 | 61.25666666666666 | 15.236666666666666 | 12.450000000000001 | 11.056666666666667 |
| 28 | 85.67666666666668 | 4.88 | 6.2 | 3.2433333333333336 |
| 42 | 70.38333333333334 | 7.043333333333334 | 13.709999999999999 | 8.863333333333333 |
| 49 | 61.01333333333332 | 12.576666666666668 | 13.5 | 12.910000000000002 |
| 70 | 72.74666666666666 | 5.45 | 9.75 | 12.053333333333333 |
| 84 | 73.34000000000002 | 3.473333333333333 | 14.530000000000001 | 8.656666666666666 |
| 98 | 68.24666666666666 | 4.3999999999999995 | 13.910000000000002 | 13.443333333333333 |
### Chart:
| Category | CD25−CD69−CD4+ T cells (%) | CD25−CD69+CD4+ T cells (%) | CD25+CD69−CD4+ T cells (%) | CD25+CD69+CD4+ T cells (%) |
|---|---|---|---|---|
| -14 | 94.11333333333334 | 0.6833333333333332 | 4.2299999999999995 | 0.9733333333333333 |
| 7 | 61.50666666666667 | 14.686666666666667 | 14.513333333333334 | 9.293333333333333 |
| 14 | 43.47666666666666 | 24.366666666666664 | 8.229999999999999 | 23.926666666666666 |
| 28 | 77.92666666666666 | 4.283333333333334 | 7.903333333333333 | 9.886666666666667 |
| 42 | 70.3 | 7.083333333333333 | 13.596666666666666 | 9.020000000000001 |
| 49 | 51.596666666666664 | 2.2633333333333336 | 30.023333333333337 | 16.116666666666664 |
| 70 | 69.31333333333333 | 8.436666666666667 | 10.266666666666667 | 11.983333333333333 |
| 84 | 71.83333333333333 | 3.34 | 18.14666666666667 | 6.68 |
| 98 | 65.82000000000001 | 4.4799999999999995 | 15.876666666666665 | 13.823333333333332 |
### Chart:
| Category | CD25−CD69−CD4+ T cells (%) | CD25−CD69+CD4+ T cells (%) | CD25+CD69−CD4+ T cells (%) | CD25+CD69+CD4+ T cells (%) |
|---|---|---|---|---|
| -14 | 74.49333333333333 | 4.726666666666667 | 11.99 | 8.790000000000001 |
| 7 | 37.25333333333333 | 8.65 | 11.873333333333335 | 42.22333333333333 |
| 14 | 55.10999999999999 | 14.339999999999998 | 10.356666666666667 | 20.193333333333335 |
| 28 | 67.87333333333333 | 5.266666666666667 | 15.266666666666666 | 11.593333333333334 |
| 42 | 69.45333333333333 | 5.646666666666666 | 18.470000000000002 | 6.43 |
| 49 | 54.12 | 9.386666666666665 | 21.939999999999998 | 14.553333333333333 |
| 70 | 60.19333333333333 | 9.653333333333334 | 14.08 | 16.073333333333334 |
| 84 | 62.30666666666668 | 5.586666666666666 | 19.016666666666666 | 13.089999999999998 |
| 98 | 57.78666666666667 | 3.33 | 21.903333333333332 | 16.98 |
### Chart:
| Category | CD25−CD69−CD4+ T cells (%) | CD25−CD69+CD4+ T cells (%) | CD25+CD69−CD4+ T cells (%) | CD25+CD69+CD4+ T cells (%) |
|---|---|---|---|---|
| -14 | 94.27 | 0.7366666666666667 | 3.736666666666667 | 1.2566666666666666 |
| 7 | 87.38999999999999 | 3.97 | 5.263333333333333 | 3.3766666666666665 |
| 14 | 74.97333333333334 | 7.426666666666667 | 9.383333333333333 | 8.216666666666667 |
| 28 | 85.15333333333332 | 5.956666666666666 | 5.026666666666666 | 3.8633333333333333 |
| 42 | 27.13666666666667 | 6.489999999999999 | 56.96 | 9.413333333333332 |
| 49 | 81.82333333333334 | 4.883333333333334 | 6.359999999999999 | 6.933333333333333 |
| 70 | 72.31333333333333 | 5.34 | 13.36 | 8.986666666666666 |
| 84 | 66.79666666666667 | 8.836666666666666 | 7.933333333333334 | 16.433333333333334 |
| 98 | 68.24666666666667 | 5.09 | 13.199999999999998 | 13.463333333333333 |
### Chart:
| Category | CD25−CD69−CD4+ T cells (%) | CD25−CD69+CD4+ T cells (%) | CD25+CD69−CD4+ T cells (%) | CD25+CD69+CD4+ T cells (%) |
|---|---|---|---|---|
| -14 | 71.46 | 4.05 | 15.08 | 9.410000000000002 |
| 7 | 41.56999999999999 | 6.633333333333333 | 27.293333333333333 | 24.50333333333333 |
| 14 | 64.63333333333333 | 10.0 | 15.286666666666667 | 10.08 |
| 28 | 45.72666666666667 | 6.0200000000000005 | 23.330000000000002 | 24.923333333333332 |
| 42 | 23.74666666666667 | 7.083333333333333 | 34.96666666666667 | 34.203333333333326 |
| 49 | 58.10666666666666 | 7.426666666666667 | 20.73 | 13.736666666666666 |
| 70 | 49.120000000000005 | 7.8933333333333335 | 11.526666666666666 | 31.459999999999997 |
| 84 | 40.13666666666666 | 7.02 | 17.71666666666667 | 35.126666666666665 |
| 98 | 53.17000000000001 | 4.256666666666667 | 20.72 | 21.853333333333335 |
### Chart:
| Category | CD25−CD69−CD4+ T cells (%) | CD25−CD69+CD4+ T cells (%) | CD25+CD69−CD4+ T cells (%) | CD25+CD69+CD4+ T cells (%) |
|---|---|---|---|---|
| -14 | 67.03 | 4.18 | 14.833333333333334 | 13.956666666666669 |
| 7 | 24.033333333333335 | 10.6 | 12.823333333333332 | 52.54333333333333 |
| 14 | 48.29333333333333 | 9.069999999999999 | 21.423333333333332 | 21.213333333333335 |
| 28 | 43.78666666666667 | 9.623333333333333 | 9.596666666666666 | 36.99333333333333 |
| 42 | 59.10666666666666 | 4.913333333333333 | 20.363333333333333 | 15.616666666666667 |
| 49 | 34.623333333333335 | 7.69 | 29.16 | 28.526666666666667 |
| 70 | 17.09333333333333 | 6.823333333333334 | 9.516666666666666 | 66.56666666666666 |
| 84 | 15.91666666666667 | 2.2366666666666664 | 12.183333333333332 | 69.66333333333334 |
| 98 | 10.84666666666667 | 0.8866666666666667 | 14.326666666666668 | 73.94 |
### Chart:
| Category | CD25−CD69−CD4+ T cells (%) | CD25−CD69+CD4+ T cells (%) | CD25+CD69−CD4+ T cells (%) | CD25+CD69+CD4+ T cells (%) |
|---|---|---|---|---|
| -14 | 70.83 | 6.57 | 15.003333333333332 | 7.596666666666667 |
| 7 | 46.52 | 12.836666666666666 | 12.799999999999999 | 27.843333333333334 |
| 14 | 25.639999999999997 | 25.676666666666666 | 9.32 | 39.36333333333334 |
| 28 | 44.56333333333333 | 3.7866666666666666 | 35.48 | 16.17 |
| 42 | 57.97666666666667 | 15.040000000000001 | 13.563333333333333 | 13.42 |
| 49 | 42.85333333333333 | 15.593333333333334 | 20.023333333333333 | 21.53 |
| 70 | 30.51 | 7.796666666666667 | 17.133333333333333 | 44.56 |
| 84 | 70.03333333333333 | 6.06 | 12.623333333333335 | 11.283333333333333 |
| 98 | 54.9 | 5.28 | 16.040000000000003 | 23.78 |Each population in CD3+CD4+ T cells (%)
### Chart:
| Category | CD25−CD69−CD4+ T cells (%) | CD25−CD69+CD4+ T cells (%) | CD25+CD69−CD4+ T cells (%) | CD25+CD69+CD4+ T cells (%) |
|---|---|---|---|---|
| -14 | 87.69000000000001 | 0.47333333333333333 | 8.396666666666667 | 3.44 |
| 7 | 69.52333333333333 | 3.7300000000000004 | 12.543333333333331 | 14.203333333333333 |
| 14 | 65.78666666666668 | 5.553333333333334 | 13.926666666666668 | 14.733333333333333 |
| 28 | 64.82666666666667 | 8.696666666666665 | 10.333333333333334 | 16.143333333333334 |
| 42 | 64.70333333333333 | 4.72 | 17.80666666666667 | 12.770000000000001 |
| 49 | 57.416666666666664 | 8.479999999999999 | 14.023333333333333 | 20.08 |
| 70 | 54.99666666666667 | 10.216666666666667 | 9.956666666666665 | 24.83 |
| 84 | 63.23333333333333 | 6.033333333333332 | 11.936666666666667 | 18.796666666666667 |
| 98 | 68.55333333333333 | 3.033333333333333 | 15.946666666666667 | 12.466666666666667 |
### Chart:
| Category | CD25−CD69−CD4+ T cells (%) | CD25−CD69+CD4+ T cells (%) | CD25+CD69−CD4+ T cells (%) | CD25+CD69+CD4+ T cells (%) |
|---|---|---|---|---|
| -14 | 86.65000000000002 | 1.9766666666666666 | 8.396666666666667 | 2.9766666666666666 |
| 7 | 79.11666666666666 | 6.663333333333333 | 8.673333333333332 | 5.546666666666667 |
| 14 | 50.38666666666666 | 21.023333333333337 | 8.216666666666667 | 20.37333333333333 |
| 28 | 66.18333333333334 | 11.356666666666667 | 9.113333333333332 | 13.346666666666666 |
| 42 | 61.27666666666667 | 12.533333333333333 | 13.79 | 12.4 |
| 49 | 60.32333333333332 | 13.213333333333333 | 13.18 | 13.283333333333333 |
| 70 | 62.54333333333333 | 9.286666666666667 | 8.863333333333333 | 19.30666666666667 |
| 84 | 70.63 | 6.06 | 12.086666666666666 | 11.223333333333334 |
| 98 | 71.93 | 3.1966666666666668 | 14.756666666666666 | 10.116666666666665 |
### Chart:
| Category | CD25−CD69−CD4+ T cells (%) | CD25−CD69+CD4+ T cells (%) | CD25+CD69−CD4+ T cells (%) | CD25+CD69+CD4+ T cells (%) |
|---|---|---|---|---|
| -14 | 82.89 | 3.14 | 8.246666666666668 | 5.723333333333334 |
| 7 | 64.81333333333333 | 11.39 | 10.806666666666667 | 12.99 |
| 14 | 68.39666666666669 | 11.956666666666669 | 8.913333333333334 | 10.733333333333334 |
| 28 | 69.94666666666667 | 7.963333333333334 | 13.516666666666666 | 8.573333333333332 |
| 42 | 74.61 | 3.7300000000000004 | 18.33 | 3.33 |
| 49 | 68.45666666666666 | 8.673333333333334 | 15.83 | 7.04 |
| 70 | 66.10000000000001 | 14.030000000000001 | 12.26 | 7.609999999999999 |
| 84 | 83.94 | 3.1033333333333335 | 9.459999999999999 | 3.4966666666666666 |
| 98 | 75.33 | 2.8466666666666662 | 16.83 | 4.993333333333333 |
### Chart:
| Category | CD25−CD69−CD4+ T cells (%) | CD25−CD69+CD4+ T cells (%) | CD25+CD69−CD4+ T cells (%) | CD25+CD69+CD4+ T cells (%) |
|---|---|---|---|---|
| -14 | 79.57333333333334 | 1.55 | 13.813333333333333 | 5.0633333333333335 |
| 7 | 39.606666666666676 | 6.02 | 28.830000000000002 | 25.543333333333333 |
| 14 | 48.29333333333333 | 9.069999999999999 | 21.423333333333332 | 21.213333333333335 |
| 28 | 76.90999999999998 | 1.5666666666666664 | 14.593333333333334 | 6.93 |
| 42 | 56.01666666666667 | 10.006666666666666 | 18.363333333333333 | 15.613333333333335 |
| 49 | 56.99 | 11.89 | 15.553333333333333 | 15.566666666666668 |
| 70 | 39.199999999999996 | 10.729999999999999 | 13.613333333333335 | 36.45666666666667 |
| 84 | 44.30666666666667 | 10.56 | 19.689999999999998 | 25.44333333333333 |
| 98 | 65.51333333333334 | 2.15 | 23.25333333333333 | 9.083333333333334 |Days post-first immunization
Figure S5. Kinetics of activation status of T cells before and after vaccination.
(A, B) Percentages of CD25+CD69+, CD25+CD69−, CD25−CD69+, and CD25−CD69− cells in CD3+CD4+ T cells in PBMCs stimulated with BRSV (A) and BVDV-1 antigen (B) in the individual animals of V and VA groups.

## Slide 6
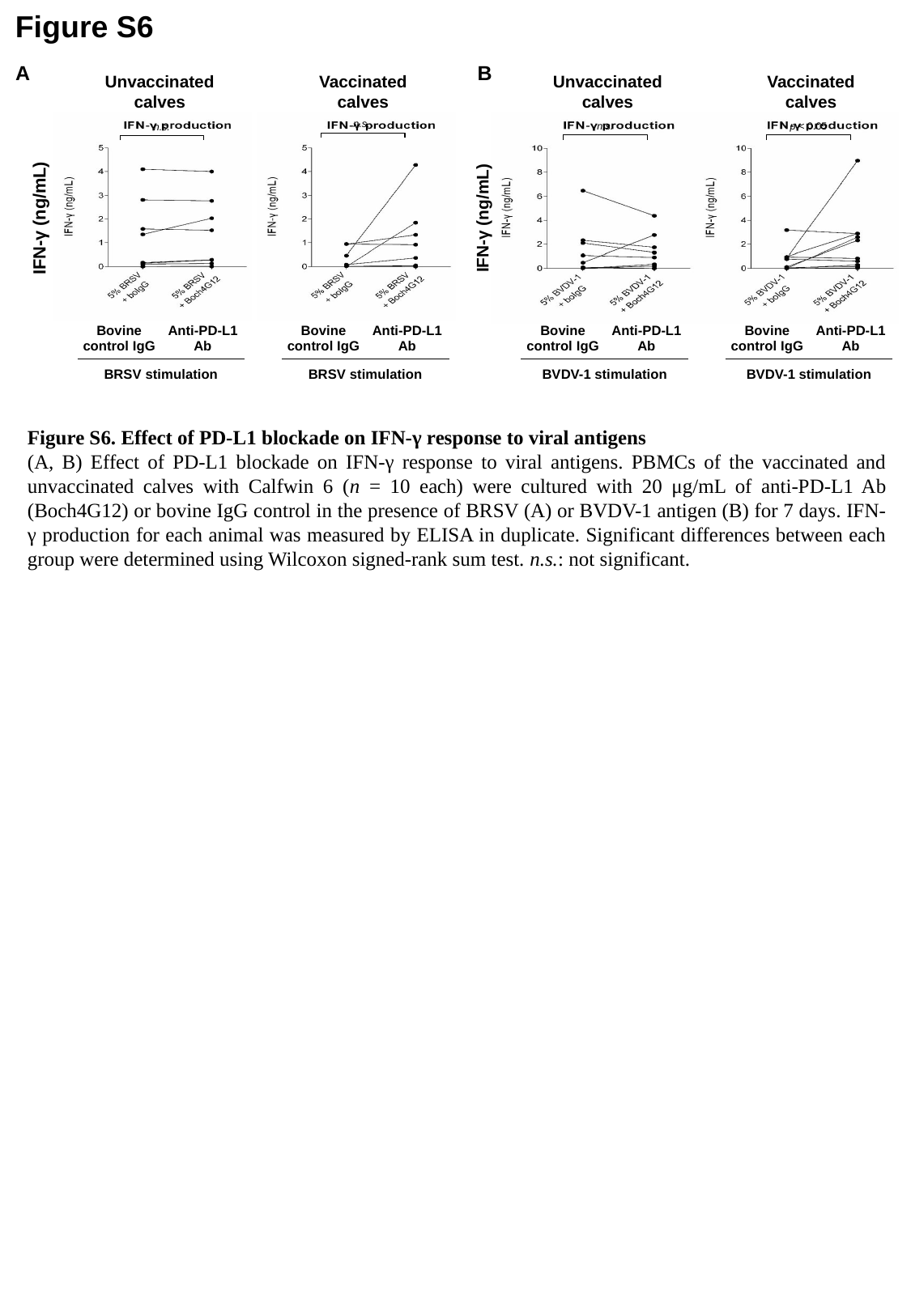

Figure S6
A
Unvaccinated
calves
Vaccinated
calves
IFN-γ (ng/mL)
n.s.
n.s.
B
Unvaccinated
calves
Vaccinated
calves
IFN-γ (ng/mL)
n.s.
p < 0.05
| Bovine control IgG | Anti-PD-L1 Ab |
| --- | --- |
| BRSV stimulation | |
| Bovine control IgG | Anti-PD-L1 Ab |
| --- | --- |
| BRSV stimulation | |
| Bovine control IgG | Anti-PD-L1 Ab |
| --- | --- |
| BVDV-1 stimulation | |
| Bovine control IgG | Anti-PD-L1 Ab |
| --- | --- |
| BVDV-1 stimulation | |
Figure S6. Effect of PD-L1 blockade on IFN-γ response to viral antigens
(A, B) Effect of PD-L1 blockade on IFN-γ response to viral antigens. PBMCs of the vaccinated and unvaccinated calves with Calfwin 6 (n = 10 each) were cultured with 20 μg/mL of anti-PD-L1 Ab (Boch4G12) or bovine IgG control in the presence of BRSV (A) or BVDV-1 antigen (B) for 7 days. IFN-γ production for each animal was measured by ELISA in duplicate. Significant differences between each group were determined using Wilcoxon signed-rank sum test. n.s.: not significant.
